# Phytochemical Properties and Antioxidant Activities of Extracts from Wild Blueberries and Lingonberries

**DOI:** 10.1007/s11130-017-0640-3

**Published:** 2017-11-14

**Authors:** Paulina Dróżdż, Vaida Šėžienė, Krystyna Pyrzynska

**Affiliations:** 1 Laboratory of Natural Environment Chemistry, Forest Research Institute, Sękocin Stary, Poland; 2 Ecology Department, Lithuanian Research Centre for Agriculture and Forestry, Kaunas Distr., Lithuania; 3 Department of Chemistry, University of Warsaw, Pasteura 1, 02-093 Warsaw, Poland

**Keywords:** Blueberry, Lingonberry, Polyphenols, Anthocyanins, Antioxidant activity

## Abstract

Among *Vaccinium* species, blueberries *(Vaccinium myrtillus* L.) and lingonberries (*Vaccinium* vitis-idaea) are popular in the human diet. In this study, total phenolic, total flavonoid and total monomeric anthocyanin contents in the ethanol-water extracts of blueberry and lingonberry fruits grown wild in the forests in the central region of Poland were assayed. Antioxidant activities of the extracts from each plant were also evaluated for scavenging ability on 1,1-diphenyl-2-picrylhydrazyl (DPPH) radicals and reducing power by cupric reducing antioxidant capacity (CUPRAC) method. Total phenolics in the blueberry extracts ranged from 4.58 to 5.28 mg GAE CE/g fw. The extracts from lingonberry fruits contained higher total contents of phenolic compounds (5.82–7.60 mg GAE/g fw) as well as total flavonoids (5.22–6.47 μmol CE/g fw) than those from blueberries (3.74–4.18 μmol CE/g fw). For the total monomeric anthocyanin contents, the blueberry extracts presented significantly higher values (3.01–3.93 mg cyanidin-3-glucoside (C3G) equivalent/g fw) in comparison to the lingonberry extracts (0.32–0.47). Blueberry extracts exhibited higher antioxidant activity measured by both assays in comparison to lingonberry extracts. Water extracts from fresh and dried fruits also exhibited significant antioxidant activities for both types of berries. Considering the health benefits that have been associated with polyphenolic consumption, these fruits could appear as a good source of this group of phytochemical compounds for their direct consumption or their use as ingredients for the design of new food products or food supplements.

**Electronic supplementary material:**

The online version of this article (10.1007/s11130-017-0640-3) contains supplementary material, which is available to authorized users.

## Introduction

Increased consumption of fruits and vegetables is recommended in dietary guidelines worldwide as they are rich in nutrients and phytochemicals. Small berry fruits are consumed because of their attractive colour and special taste, and are considered one of the richest sources of natural antioxidants [1, 2]. Among *Vaccinium* species, blueberry (*Vaccinium myrtillus* L.) and lingonberries (*Vaccinium* vitis-idaea) are popular in the human diet either fresh or in processed forms [3]. Fruits are mostly collected from wild plants growing on publicly accessible lands and you can buy them at the local markets. Their consumption has been linked to the prevention of some chronic and degenerative diseases and the most significant health benefits are ascribed to phenolic compounds and vitamin C [4–6]. These berries also contain other bioactive substances, such as folate, potassium and soluble fiber. *Vaccinium myrtillus* fruits have been used in the traditional medicine internally (as tea or liqueur) for treatment of disorders of the gastrointestinal tract and diabetes.

Polyphenols such as flavonoids, and derivatives of hydroxycinnamic and hydroxybenzoic acids, anthocyanins, and procyanidins are found in particularly high concentration in various berries [7–11]. The differences in phenolic profiles in blueberry or lingberry fruits have been linked to the species and cultivar as well as to growing conditions, maturity at harvest and processing operations. Polyphenolic compounds exhibit a wide range of biological effects, including antibacterial, anti-inflammatory, anti-allergic and anti-thrombotic actions [12]. These beneficial effects are mainly due to their antioxidant activity as they can act as reducing agents, hydrogen donors, singlet oxygen quenchers as well as chelators of metal ions, preventing metal catalyzed formation of free radicals [13, 14].

The aim of this study was to evaluate the major classes of polyphenolic compounds (total phenolics, flavonoids and anthocyanins) from blueberry and lingonberry fruits grown wild in the forest in the central region of Poland. The limited area was selected in order to have uniform climatic conditions as well as altitude. Antioxidant activities of the extracts prepared from fresh and dried berries were also evaluated for scavenging ability on 1,1-diphenyl-2-picrylhydrazyl (DPPH) radicals and reducing power by cupric reducing antioxidant capacity (CUPRAC) method.

## Material and Methods

### Chemicals and Fruit Samples

1,1-diphenyl-2-picrylhydrazyl (DPPH), Folin-Ciocalteau phenol reagent, trolox, catechin, and gallic acid were purchased from Sigma-Aldrich. Cyanidin-3-glucoside was obtained from Extrasynthese (Lyon, France). All other reagents were of analytical purity (Merck). Ethanol for extraction of phenolics was acquired from Merck.

Fruits of wild growing blueberries and lingonberries were collected at the same places in pine forest during September of 2016, in three different locations in the Mazovia region. Coordinates for sample from Wyszków N 52° 41′, E 21° 29, from Ostrowia N 52° 49′, E 21° 45′ and from Chojnów N 52° 01′, E 21° 06′. Fruits were stored at −20 °C, they were thawed at refrigerator temperature (~4 °C) and homogenized by blender for extraction and analysis. Drying of fruits was performed in laboratory dryer at 60 °C for 24 h.

### Preparation of the Extracts

For extraction from fresh or dried fruits, 700 mg of the homogenized material was shaken with 25 mL of deionized water or ethanol-water (60:40, *v*/v) solution for 20 min at 55 °C. Then, the extracts were filtered through Whatman no.1 filter paper. For a given sample (appropriate fresh or dried fruits) three independent extractions using water or hydroalcoholic solution were carried out.

### Determination of Total Phenolics

Total phenolic content of extracts was assessed by using the Folin–Ciocalteu (FC) phenol reagent method [15]. 0.1 mL of extract was mixed with 0.1 mL of FC reagent and 0.9 mL of water. After 5 min, 1 mL of 7% (*w*/*v*) Na_2_CO_3_ and 0.4 mL of water were added. The extracts were mixed and allowed to stand for 30 min before measuring the absorbance on a spectrophotometer (PerkinElmer, UV–visible Lambda Bio 20) at 765 nm. A mixture of water and reagents was used as a blank. Total phenolic content was expressed as gallic acid equivalents (GAE) in mg g^−1^ fresh weight of fruit. Each analysis was done in three repetitions.

### Determination of Flavonoid Content

Total flavonoid content was determined using a spectrophotometric method based on formation of their complexes with Al(III) [16]. 1 mL of a sample was mixed with 0.3 mL of NaNO_2_ (5%, *w*/*v*) and after 5 min 0.5 mL of AlCl_3_ (2%, w/v) was added. A sample was mixed and six minutes later was neutralized with 0.5 mL of 1 mol/L NaOH solution. The mixture was left for 10 min at room temperature and then absorbance was measured at 510 nm. The results were expressed as catechin equivalent (CE) in μmol per gram fresh weight of fruit. Absorbance was measured in three replications.

### Determination of Monomeric Anthocyanins

The total monomeric anthocyanin content was determined using pH-differential method [17]. 1 mL of the extract was poured into two separate volumetric flasks. One of them was filled with KCl solution (pH 1) and the second with CH_3_COONa (pH 4.5) and these two solutions were left for 30 min at room temperature. Finally, the absorbance of both samples was recorded at wavelengths of 520 and 700 nm. The results were expressed as mg of cyanidin-3-glucoside (C3G) per gram of fresh sample. Each analysis was done in three repetitions.

### Free Radical Scavenging Activity

The DPPH assay was applied to estimate the radical-scavenging ability of the fruit extracts [18]. 0.1 mL of a given extract was mixed with 2.4 mL of DPPH solution (9 × 10^−5^ mol/L) in methanol and after 30 min the change of absorbance at 518 nm was recorded. Trolox, a vitamin E analogue, was used as the standard solution and the results were expressed in trolox equivalent (TE) mmol per gram of fresh fruits.

### Cupric Ion Reducing Capability

For assessing cupric reducing ability (CUPRAC), the assay described by Apak et al. [19] was adapted. 1 mL of CuCl_2_ solution (1 × 10^−2^ mol/L) was mixed with 1 mL of neocuproine alcoholic solution (7.5 × 10^−3^ mol/L) and 1 mL of 1 mol/L acetate ammonium solution, followed by mixing 0.5 mL of a given extract and 0.6 mL of water. The mixture was incubated in a water bath at a temperature of 50 °C for 20 min. The absorbance against the reagent blank was measured after 30 min at 450 nm. The results are expressed as trolox equivalent (TE) in mmol/L per gram of fresh fruits.

## Results and Discussion

For the extraction of polyphenolic compounds from studied berry fruits water and ethanol-water solution (60:40, *v*/v) were chosen. The water extracts of berries are important from the household brewing and consumption [20–22]. For incorporation into a functional beverage, aqueous alcohol have been used with the aim to maximizing the active ingredients [23–25]. As previously observed [26, 27], an increase in the extraction temperature can promote higher phenolics solubility from plant materials, but also heat treatment may be responsible for their partial destruction. Thus, in the preliminary experiments the stability of phenolic compounds present in studied fruits of berries was checked at elevated temperature during extraction process. A higher temperature (55 °C) led to an increased extraction yield of total phenolics from berries by 10–20% in comparison to 20 °C temperature (data not shown). It suggested that berry phenolics are relatively stable under higher temperature conditions during 20 min of extraction. Our results are in accordance with Arancibia-Avila et al. [28], who found that berries subjected to thermal processing not more than 20 min maximally preserved their bioactivity.

The results for total phenolic and total flavonoid contents in the studied extracts are shown in Table 1. For ethanol-water extracts higher results were obtained in comparison to water infusions as the solubility of phenolics is higher in alcohols. The total phenolics in the blueberry hydroalcoholic extracts ranged from 4.58 to 5.28 mg GAE/g. The extracts from lingonberry fruits contained higher total contents of phenolic compounds (5.82–7.60 mg GAE/g) as well as total flavonoids (5.22–6.47 μmol CE/g) than those from blueberries (3.74–4.18 μmol CE/g). The results of one-way ANOVA followed by Turkey’s test indicated that the total flavonoid contents obtained for all blueberry samples were statistically similar (*p* < 0.05), while for lingonberry samples collected from the same places vary significantly. The polyphenol classification proposed by Vasco et al. [29] using low (< 1 mg GAE/g), medium (1–5 mg GAE/g) and high (> 5 mg GAE/g) denominations, indicates that both our berry samples are a good source of these compounds.

**Table 1:** Total phenolics, flavonoids and monomeric anthocyanins content in blueberry and lingonberry extracts

Locality of collection	Total phenolics (mg GAE g^−1^ fw)	Total flavonoids (μmol CE g^−1^ fw)	Total anthocyanins (mg C3G g^−1^ fw)	Total phenolics (mg GAE g^−1^ fw)	Total flavonoids (μmol CE g^−1^ fw)	Total anthocyanins (mg C3G g^−1^ fw)
Blueberries: water extracts				Blueberries: ethanol-water extracts		
Wyszków	4.57 ± 0.17^a^	1.94 ± 0.11^a^	3.44 ± 0.17^a^	5.28 ± 0.07^a^	3.74 ± 0.07^a^	3.93 ± 0.40^a^
Ostrowia	3.90 ± 0.15^b^	2.21 ± 0.04^b^	2.79 ± 0.22^b^	5.26 ± 0.16^a^	4.08 ± 0.17^a^	3.01 ± 0.22^b^
Chojnów	3.66 ± 0.02^c^	1.63 ± 0.07^c^	2.44 ± 0.05^b^	4.58 ± 0.27^b^	4.18 ± 0.19^a^	3.23 ± 0.14^b^
Lingonberries: water extracts				Lingonberries: ethanol-water extracts		
Wyszków	4.36 ± 0.18^a^	2.55 ± 0.07^a^	0.38 ± 0.01^a^	5.82 ± 0.18^a^	5.22 ± 0.11^a^	0.47 ± 0.01^a^
Ostrowia	6.06 ± 0.16^b^	3.38 ± 0.19^b^	0.34 ± 0.02^a^	7.10 ± 0.05^b^	6.47 ± 0.15^b^	0.35 ± 0.01^b^
Chojnów	6.36 ± 0.07^b^	3.53 ± 0.21^b^	0.41 ± 0.03^a^	7.60 ± 0.27^c^	5.79 ± 0.11^c^	0.42 ± 0.02^c^

Results expressed as mean ± SD (*n* = 3)

Different letters in each column represent significant differences (*p* < 0.05)

The mean concentration of phenolic compounds in lingonberries grown in a research plot in Oregon (United States) was 5.66 mg GAE/g (range 4.31–6.60 mg GAE/g) [30]. Significantly lower results (0.36–0.41 mg GAE/g) for various lingonberry extracts (methanol, ethanol, acetone, ethyl acetate and their mixture with water) were reported for fruits harvested from southern Labrador area in Canada [31]. The average total polyphenol content determined in wild blueberries collected from different localities in Slovakia was 2.86 mg GAE/g and it was 97% higher compared to highbush cultivars (1.45 mg GAE/g) [23]. The variation between the results presented in this paper and the previously published data can be explained by the influence of cultivar, ripening stage, weather and soil conditions as well as various applied extraction methods. As reported in the literature [9, 24], the main flavonoids in lingonberry extracts are flavan-3-ols (catechin and epicatechin) as well as flavonols, mainly quercetin glycosides. Presence of quercetin glycosides, together with hydroxycinnamic acids, is also characteristic for the blueberry extracts [9, 32].

For the total monomeric anthocyanin contents, the blueberry extracts presented significantly higher values (3.01–3.93 mg of cyanidin 3-glucoside equivalent/g) in comparison to the lingonberry extracts (0.35–0.47) (Table 1). Similar value (2.9 mg/g) were reported by Chorfa et al. [32] for wild blueberries collected from Lake Saint-Jean region in Ontario, Canada with the highest contribution from malvidin-3-glucoside and peonidin-3-glucoside. Garzón et al. [7] determined total anthocyanin content in blueberries native to Colombia as 3.3 mg of cyanidin 3-glucoside per gram. Fruits of five lingonberry cultivars grown in Oregon (USA), contained total anthocyanins in the range of 0.27–0.53 mg cyanidin 3-glucoside equivalent per gram [17].

**Fig. 1:**
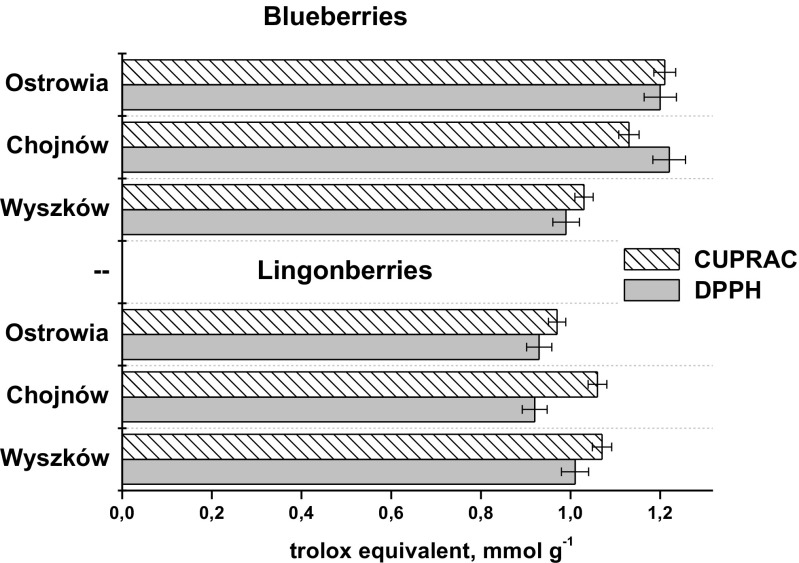
Antioxidant activities of lingonberry and blueberry extracts evaluated by CUPRAC and DPPH assays

The antioxidant activities of both berry extracts were determined by DPPH and CUPRAC assays. The DPPH assay measures the ability of the antioxidants to quench DPPH^**·**^ radicals by an electron transfer reaction, while CUPRAC method measures the reducing power of sample constituents relating to their electron transfer ability. According to our results presented in Fig. 1, studied lingonberry and blueberry fruits are good electron donors as their extracts were able to reduce the copper(II)-neocuproine chelate as well as to quench DPPH^**·**^ radicals. Blueberry extracts exhibited higher antioxidant activity measured by both assays in comparison to lingonberry extracts. Their higher content of total anthocyanins (Table 1) are most likely to contribute to the radical scavenging and antioxidant activity. Lingonberries with high amounts of hydroxycinnamates such as chlorogenic acid benefit from their antioxidant effect [24].

During the winter when fresh fruits are not available, dried berries can be used in the form of tea or they are added to cereal or snacks as a good source of antioxidants in concentrated form. Extracts of blueberry have shown a protective effect on visual function during retinal inflammation [33] and antiproliferative activity after *in vivo* treatment of human cancer cell lines [34]. Thus, it was interesting to check the antioxidant activity of water and hydroalcoholic extracts prepared also from dried berry fruits under study. Drying of these fruits was performed at 60 °C for 24 h and extraction was done for 20 min using 20 mL of freshly boiled distilled water or ethanol-water (60:40, *v*/v) solution.

Figure 1S presents the kinetic curves of scavenged DPPH radicals by different extracts of fresh dried berries. The sample collected at location Wyszków were taken as the example. Note that extracts from dried fruits were diluted 10-fold for measurement. A fast initial decrease of absorbance of DPPH radical followed by slow subsequent disappearance of this reagent can be observed from presented plots. According to Villaño et al. [35], this fast step essentially refers to the electron-transfer process from a phenol molecule or its phenoxide anion to DPPH free radicals while the subsequent decay reflects the remaining activity of the oxidation-degradation products. As it was expected, for ethanol-water extracts higher results were obtained in comparison to water infusions as the solubility of phenolic antioxidants is higher in alcohols. However, water extracts also exhibited significant antioxidant activities and could be a good source of phenolics in juices, sauces or jellies. Total phenolic contents in water extracts of fresh blueberries and lingonberries were 4.57 and 4.36 mg GE/g of fresh matter, while the values of 26.4 and 23.6 mg GE/g of dry matter were obtained for dried fruits, respectively. Dried berries had the total phenolics as well as a DPPH score much higher than their respective fresh forms due to dehydratation. Removing moisture and making fruit leather concentrated the skin and pulp, increasing the antioxidant levels in each gram of product. Thus, the consumption of fruit teas or eating dried berries as snacks can be part of a healthy lifestyle. Although phenolic composition and antioxidant activities of dried blueberry was reported before [34], there is no information available about dried lingonberries.

## Conclusions

From the results of this study can be concluded that Polish wild blueberries and lingonberries collected in Mazovia region contains substantial amounts of phenolic compounds. Free scavenging assay provided evidence that fresh and dried berries may have biomedical application in reducing body oxidative stress, a deteriorating situation arising from an increase of various reactive oxygen species. Picking berries from their native forest place provides healthy food in addition to benefits of physical activity in the fresh air.

## Electronic supplementary material

ESM 1 (DOC 136 kb)
