# Supplementary material for: Phytochemical Properties and Antioxidant Activities of Extracts from Wild Blueberries and Lingonberries
Source: Plant Foods Hum Nutr. 2017 Nov 14;72(4):360–4. doi: 10.1007/s11130-017-0640-3 (PMC5717128; doi:10.1007/s11130-017-0640-3)
Supplement: Supplementary file 1 — (DOC 136 kb) [file 11130_2017_640_MOESM1_ESM.doc]

**Supplementary Material**

**Phytochemical properties and antioxidant activities of extracts from wild blueberries**

**and lingonberries**

Paulina Dróżdż, Vaida Šėžienė**,** Krystyna Pyrzynska

**Fig. 1S.** The kinetic curves of scavenged DPPH radicals by different extracts of fresh and dried berries. The extracts from dried fruits were diluted 10-fold for measurement.
